# Mobile Technologies: Expectancy, Usage, and Acceptance of Clinical Staff and Patients at a University Medical Center

**DOI:** 10.2196/mhealth.3799

**Published:** 2014-10-21

**Authors:** Kristin Illiger, Markus Hupka, Ute von Jan, Daniel Wichelhaus, Urs-Vito Albrecht

**Affiliations:** ^1^PL Reichertz Institute for Medical InformaticsHannover Medical SchoolHannoverGermany; ^2^Faculty IVUniversity of Applied Sciences and Arts, HannoverHannoverGermany

**Keywords:** survey, mobile health, mobile apps, health care, privacy, data protection, patients, medical staff, staff attitude

## Abstract

**Background:**

Despite their increasing popularity, little is known about how users perceive mobile devices such as smartphones and tablet PCs in medical contexts. Available studies are often restricted to evaluating the success of specific interventions and do not adequately cover the users’ basic attitudes, for example, their expectations or concerns toward using mobile devices in medical settings.

**Objective:**

The objective of the study was to obtain a comprehensive picture, both from the perspective of the patients, as well as the doctors, regarding the use and acceptance of mobile devices within medical contexts in general well as the perceived challenges when introducing the technology.

**Methods:**

Doctors working at Hannover Medical School (206/1151, response 17.90%), as well as patients being admitted to this facility (213/279, utilization 76.3%) were surveyed about their acceptance and use of mobile devices in medical settings. Regarding demographics, both samples were representative of the respective study population. GNU R (version 3.1.1) was used for statistical testing. Fisher’s exact test, two-sided, alpha=.05 with Monte Carlo approximation, 2000 replicates, was applied to determine dependencies between two variables.

**Results:**

The majority of participants already own mobile devices (doctors, 168/206, 81.6%; patients, 110/213, 51.6%). For doctors, use in a professional context does not depend on age (*P*=.66), professional experience (*P*=.80), or function (*P*=.34); gender was a factor (*P*=.009), and use was more common among male (61/135, 45.2%) than female doctors (17/67, 25%). A correlation between use of mobile devices and age (*P*=.001) as well as education (*P*=.002) was seen for patients. Minor differences regarding how mobile devices are perceived in sensitive medical contexts mostly relate to data security, patients are more critical of the devices being used for storing and processing patient data; every fifth patient opposed this, but nevertheless, 4.8% of doctors (10/206) use their devices for this purpose. Both groups voiced only minor concerns about the credibility of the provided content or the technical reliability of the devices. While 8.3% of the doctors (17/206) avoided use during patient contact because they thought patients might be unfamiliar with the devices, (25/213) 11.7% of patients expressed concerns about the technology being too complicated to be used in a health context.

**Conclusions:**

Differences in how patients and doctors perceive the use of mobile devices can be attributed to age and level of education; these factors are often mentioned as contributors of the problems with (mobile) technologies. To fully realize the potential of mobile technologies in a health care context, the needs of both the elderly as well as those who are educationally disadvantaged need to be carefully addressed in all strategies relating to mobile technology in a health context.

##  Introduction

### Mobile Phones and Health Care

The widespread use of mobile devices such as smartphones and tablets, or more specifically, mobile devices able to run various types of application software (apps) does not stop at health care; apps running on such devices provide users with health information, measure their bodily functions, remind them about taking their medication, or support diagnostics. According to current findings by Tran et al (2014), in medicine, the use of mobile phones in a medical setting is increasingly coming into the focus of (international) research [1]. Studies performed in these contexts often deal with, and identify chances as well as challenges and risks of using health apps and medical apps in health care [2-4].

The use of mobile devices in daily clinical practice does not only touch on questions regarding technical feasibility, structural framework conditions, or political aspects, rather, using or refraining from using these devices should always be an individual choice of the clinicians. Based on a longitudinal study where we surveyed medical doctors working at the Hannover Medical School at two points in time, specifically the summer of 2012 and spring of 2014, we were able to confirm that the use of mobile devices in professional settings is rapidly increasing, both when collaborating with colleagues as well as when interacting with patients [5]. This increase does not only cover the increased frequency of use, but also the expansion of the areas of application where mobile devices are used. Our findings also show that there have been only marginal changes regarding concerns voiced by the staff regarding the use of smartphones and tablets. In contrast to [5], where we only evaluated how the use of mobile devices in a professional setting had changed between 2012 and 2014, this time around we also wanted to include patients. Specifically, we wanted to determine whether there are any notable differences in how patients and doctors view the use of mobile devices when it comes to using them in a health related context.

### Narrow Perspectives of Previous Studies

Many publications only consider a relatively narrow perspective, for example, the patients’ [6,7], or doctors’ [8,9] point of view, or a specific field of application [10] when looking at the challenges and potentials of using mobile devices in a clinical context. Also, these studies often only look at whether mobile devices work for a specific intervention or area of application, rather than at the general question of what makes their use attractive for potential users or which factors may keep potential users from using the devices. Thus, one may miss the chance of painting a comprehensive picture of the acceptance and use of mobile devices in medical settings, which would be necessary for letting both groups, patients as well as medical professionals, participate and benefit from mobile technical innovations during the care process. Only when including these aspects is it possible to account for the qualms doctors, as well as their patients, may have regarding the use of mobile devices in medicine.

### The Objectives

Our objective was therefore to obtain a comprehensive picture of how mobile smart devices are perceived in medical contexts, and how they are used in reality. The evaluation therefore covers not only the purposes for which both doctors as well as their patients are already using mobile smart devices, but also concerns they may have or challenges they perceive when using such technology. In this context, it was also of interest whether medical professionals have a different view with respect to the use of mobile technology or toward the perceived dangers.

## Methods

### Multi-Perspective Approach

Based on a multi-perspective approach, doctors working at Hannover Medical School, a maximum care university hospital located in northern Germany, as well as patients being admitted to this facility, were surveyed about their acceptance of mobile devices in medical settings and how they made use of this technology. This study was registered with the institutional review board of Hannover Medical School (trial number 1206-2011).

### Doctors’ Survey

Data regarding doctors were obtained based on a standardized and anonymous online survey that was performed between February 6th and March 12th, 2014. All doctors employed by Hannover Medical School during this time span were invited to participate. Following the first call for participation, two reminders were sent to those who had not yet participated. Altogether, 206 out of 1151 eligible doctors participated in the survey, corresponding to a response rate of 17.90%. The sociodemographic data of those who answered are representative of the overall population of doctors working at Hannover Medical School [11]. Table 1 describes the sociodemographic data for the sample (206/1151) we obtained.

**Table 1:** Sociodemographic data of the physicians (206/1151) who answered the survey.

Sociodemographic data of the physicians		Number of physicians who answered survey=206, n (%)
**Gender**		
	Male	135 (65.5)
	Female	67 (32.5)
	Not specified	4 (1.9)
**Age (years)**		
	18-25	1 (0.5)
	26-35	80 (38.8)
	36-45	92 (44.2)
	46-55	23 (11.2)
	56 and older	6 (2.9)
	Not specified	5 (2.4)
**Work experience (years)**		
	None	4 (1.9)
	1 up to 2	11 (5.3)
	2 up to 4	31 (15.0)
	4 up to 6	26 (12.6)
	6 up to 10	35 (17.0)
	10 up to 20	69 (33.5)
	20 up to 30	20 (9.7)
	30 and longer	7 (3.4)
	Not specified	3 (1.5)
**Role**		
	Chief physician	1 (0.5)
	Consultant	75 (36.4)
	Attending	25 (12.1)
	Junior doctor	99 (48.1)
	Not specified	6 (2.9)

### Patients’ Survey

A systematic random sample of adult patients presenting at the central admissions point of Hannover Medical School was used to perform the patient specific survey. Patients below 18 years of age were not included in the survey, since at Hannover Medical School, children and adolescents are not admitted via the central admissions point, but through the separate admissions point of the pediatric clinic. The survey was performed on five days (over periods of two to four hours per day) between November 12th, 2013 and December 10th, 2013. During the survey, altogether N=558 patients entered the central admissions point and every second patient (279/558) was asked whether he or she was willing to participate in the survey. The survey was performed in the form of oral interviews, and the survey personnel consisted of 7 students of the University of Applied Sciences and Arts at Hannover who had been instructed about how to perform the interviews; specifically how to go through the questions; to provide explanations when needed, but to refrain from using suggestive explanations; and to avoid touching on personal matters such as specifics of a patient’s condition. Altogether, 213/279 individuals were willing to participate and were thus included in the survey, corresponding to a utilization rate of 76.3%. Main reasons for not participating were language problems, as well as patients being called in too soon to finish the survey.

The demographics (age, gender, etc) of the patients included within the survey (Table 2) were comparable to the demographics of the overall patient population (provided in anonymized form by the hospital’s administration) encountered at the admissions point during the survey.

**Table 2:** Demographics of the patients (n=213) participating in the survey.

Sociodemographic data of the patients		n (%)
**Gender**		
	Male	115 (54.0)
	Female	85 (39.9)
	Not specified	13 (6.1)
**Age (years)**		
	18-25	18 (8.5)
	26-35	30 (14.1)
	36-45	26 (12.2)
	46-55	30 (14.1)
	56-67	47 (22.1)
	68 and above	47 (22.1)
	Not specified	15 (7.0)
**Educational level**		
	Primary school	2 (0.9)
	Lower secondary school	37 (17.4)
	Intermediate secondary school	73 (34.4)
	Upper secondary school	84 (39.4)
	Not specified	17 (8)
**Condition**		
	Acute	73 (34.3)
	Chronic	123 (57.7)
	Not specified	17 (8.0)

### Instruments Used During the Survey

#### Questions Used for Doctors

Although different means were used for administering the surveys for patients and doctors, in accordance with our objectives, the design of the questionnaires used for surveying both patients and doctors was largely similar. Depending on the question, answers were either given as “yes” or “no”, as a choice between various options, or as free text answers.

The electronic questionnaire used for surveying the doctors contained 15 items (see Multimedia Appendix 1) that were relevant for the presented evaluation. Another 12 items that were also included in the questionnaire focused on a slightly different subject area and are thus not included here.

Among others, two questions dealt with whether the participant had access to a mobile device, and if so, which type of device was available. Another seven questions covered current and desired usage scenarios (including one question with free text answers where the participants could state which, if any, apps they were already using), as well as possible concerns arising from the use of mobile devices in a medical context. In addition, the participants were given an opportunity to voice their opinion or make remarks by entering text in a text entry field. They were also asked to provide information about their age and gender, as well as their professional experience (none, 1 up to 2 years, 2 up to 4 years, 4 up to 6 years, 6 up to 10 years, 10 up to 20 years, 20 up to 30 years, 30 years, and more), as well as their professional function (chief physician, consultant, attending, junior doctor). There was also one field where the participants could enter remarks.

#### Questions Used For Patients

The questionnaire employed for the oral interviews of the patients contained 17 items (see Multimedia Appendix 2). Again, the first two questions covered availability of a mobile device, as well as the type of device (if one was available). Another five items covered current and desired use of mobile devices with respect to medical and health issues. An additional five items were included to obtain information about the participants’ attitude toward mobile devices being used by their attending physicians. There were also four questions regarding age, gender, as well as the school-leaving qualification, and whether the patients were seeking help for an acute or a chronic condition. This was of interest since various factors, including but not limited to, age and educational level may influence access to mobile technologies, as well as the level of competence individuals exhibit when dealing with such technologies [12-14]. Also, patients with chronic conditions are often rated as “experts” when it comes to their condition, dealing with the condition on a daily basis, medication for their condition, etc [15,16]. In this light, we were therefore interested whether, and, if yes how, the answers provided regarding use and appraisal of mobile devices in a medical context differed between patients presenting with acute or chronic conditions. According to [15], chronic conditions are defined as the “...result of an ongoing process of degenerative changes in the somatic or psychological status”. Nevertheless, in literature, there is no uniformly recognized definition of the amount of time needed to make an acute condition chronic, and numbers often range between 3 and 6 months [17,18]. In the context of our evaluation, we decided to use a duration of at least 6 months to define a “chronic condition”.

### Statistical Methods

GNU R (version 3.1.1) was used for statistical evaluation. Fisher’s exact test, two-sided, alpha=.05 with Monte Carlo approximation, 2000 replicates, was applied to determine dependencies between two variables. For the patient survey, calculations were performed following the assumptions outlined in section “Instruments”, specifically for age, gender, education, and disease status. For evaluating the data obtained from the doctors’ survey, regarding professional function, the values obtained for chief physicians and consultants were aggregated since there was a low response rate from chief physicians. In addition, regarding the educational level of the patients, those who had attended primary school or lower secondary school were also aggregated since the number of patients who had only finished primary school was low.

## Results

### Doctors’ Survey

At the time of our second survey in 2014, the majority of doctors who had answered owned one or more mobile devices (168/206, 81.6%). There were (78/206) 37.9% of them that admitted to using the device for work purposes (without knowing how often they did so, since we did not ask for how frequently they used the devices). Another (77/206) 37.4% deem it highly probable that they would be using such a device for work in the future, (46/206) 22.3% believe this to be unlikely in the near future. Whether a mobile device is already used in a professional context does not depend on age (*P=*.66), professional experience (*P=*.80), or professional function (*P=*.34). However, this changes when looking at gender versus professional use of mobile devices (*P=*.009), with (61/135) 45.2%, use is more common among male participants than among female doctors, where only (17/67) 25% of those who had answered the survey were already using mobile devices for their work.

**Figure 1:**
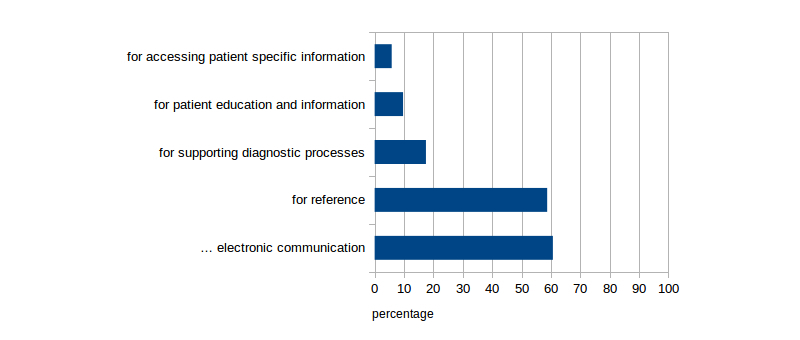
Activities the mobile devices were used for by the participating physicians.

The majority of doctors who were using at least one mobile device for work had purchased or received the device(s) privately; only 12 had received a device from their employer. While a larger number of participants owned an iPhone, Android based phone, or Blackberry (158/168, admitting to professional use of mobile devices, 69/158), there is also a considerable number of those who own an Android or iOS based tablet PC (80/168, admitting to professional use of mobile devices, 46/80). Other mobile phones or tablets are negligible (7/168), and many doctors (69/168) own more than one device. Main application areas were sending emails (125/206, 60.7%) or looking up medical information (121/206, 58.7%). Almost every fifth participant admitted to using their devices during contact with patients, for example, for showing information to their patients or as a diagnostic aid (Figure 1 shows this information).

The types of apps that had been installed by the participating physicians, primarily, also mirror these activities; the interviewees stated that they used apps for literature searches, medication databases, or apps provided by medical journals. In the free text answers, there were two mentions of professional online networks for doctors where users can discuss specific cases.

Concerns regarding the use of mobile devices during physician-patient encounters were mainly voiced with respect to the safety of patient data (129/206, 62.6%), hygiene (80/206, 38.8%), and credibility of the provided content (67/206, 32.5%). For these three aspects, there were no differences between those already using mobile devices in the course of their work and those not (yet) using mobile devices for professional purposes. In contrast, the latter group worries more about the technical reliability of the devices than those already using them, and the significance between both groups is significant (*P=*.03). Only a few physicians were concerned about patients not having access to such technologies or being unfamiliar with it (17/206, 8.2%). Too much time being required for familiarizing oneself with mobile technology or lack of interest were of little consequence for the use of mobile devices in a professional context (6/206, 2.9%).

### Patients’ Survey

**Figure 2:**
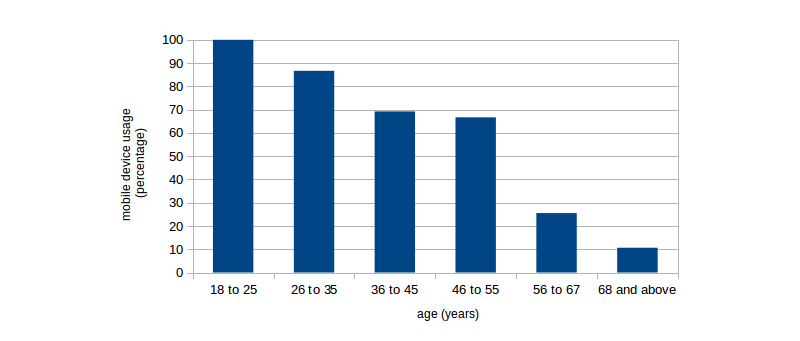
Availability/ownership of mobile devices within different age cohorts.

Over half of the participating patients (110/213, 51.6%) already use a smartphone and/or a tablet PC (mainly iPhones or Android devices, iPhone; 30/110, 27.3%; Android phones; 66/110, 60.0%). Availability and use of mobile devices presents itself differently within different age groups (Figure 2 shows this information).

Apart from the association between age and ownership/availability of a mobile device (*P=*.001), there is also a significant correlation between education and ownership of such a device (*P=*.002), also see Table 3.

**Table 3:** Ownership of a mobile device versus educational level.

School-leaving qualification	Absolute number of participants who own a mobile device	Percentage per school-leaving qualification, % (n)
Primary or lower secondary school	11	30 (11/37)
Intermediate secondary school	39	53 (39/73)
Upper secondary school	52	62 (52/84)
Not specified	23	

### Patients’ Use of Mobile Devices

There were (53/110) 48.2% of patients who owned a mobile device, and provided any information about their health related usage of the device, that stated that they were using it for looking up health related information, and for managing their own health data. In this respect, chronically ill patients do not differ from those presenting with an acute condition. Main activities where the devices are used in a health context are the search for information about symptoms and specific health conditions (43/53, 81%), or specific diagnostic methods as well as treatments (36/53, 68%), as well as looking up desired and adverse effects of specific medications, medical devices, and therapies (28/53, 53%). The devices are also often employed for searching for doctors (39/53, 74%) or pharmacies (37/53, 70%). Less often do those surveyed use their devices for communicating (via email, chat, etc) with their doctors (14/53, 26%), insurance companies (12/53, 23%), or other health care service providers (10/53, 19%).

**Figure 3:**
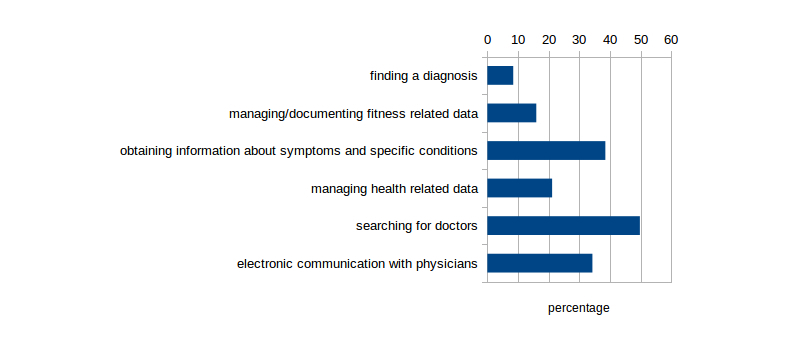
Health related activities patients would like to perform with their mobile devices.

When asked about what they would like to use a mobile device for, independent of whether they already own and use such a device or not, searching for doctors (106/213, 49.8%) and pharmacies (76/213, 35.7%), as well as looking up information about symptoms and specific health conditions are mentioned most often (82/213, 38.5%). Compared to the numbers above, communication with physicians gains importance (73/213, 34.3%). The use case patients mention least often is trying to establish a diagnosis on their own (18/203, 8.9%; Figure 3 shows this information).

Of those owning a smartphone or tablet PC, (43/110) 39.1% have one or more health related apps installed on their device. Specific apps mentioned by the participants include fitness apps (four apps), apps provided by insurers (one app), apps for obtaining the heart rate (two mentions), weight loss apps (one app), as well as vision tests (one app), and apps for specific pharmacies (five mentions). Looking at the apps mentioned by the participants, it becomes clear that the apps they mention are health apps rather than medical apps, if one follows the definition of health apps and medical apps taken from [19], where Albrecht et al recommend a differentiation between the terms “health app” and “medical app”, based on the definitions of “health” given by the World Health Organization in 1946, where health is defined as "a state of complete physical, mental, and social well-being and not merely the absence of disease or infirmity" [20]. Apps with a purpose that follow this definition of health can therefore be counted as “health apps”, and this also includes fitness and wellness related apps, and the apps mentioned by our participants clearly fall into this category.

In contrast, although apps that deal with the prevention of diseases, injuries, or support diagnostics and treatment could also be covered by the term “health apps”, they should more fittingly be labeled as “medical apps”, such apps clearly touch on areas typically covered by health care professionals, and thus, assigning the label “medical app” seems more appropriate to underline their diagnostic and therapeutic aspects [19]. None of the apps mentioned by the patients participating in our survey meets this definition of a “medical app”.

Apart from their own use behavior, we also asked whether they have any reservations about using the devices, and whether they are comfortable with doctors using mobile devices during patient-physician interactions. The majority tolerated communication related use during such interactions. There were (180/199) 90.5% (who answered this question) that consider it acceptable if doctors use the devices to illustrate something. Almost three quarters of the participants (155/197, 78.7% of those who answered this question) have no problem if doctors use a mobile device to inform themselves about their condition.

In the context of physicians using mobile devices while treating them, those surveyed were particularly worried about data protection. Roughly every fifth participant (45/202, 22.3%) did not want their doctors to save or process their individual health related data on a mobile device, whereas (145/202) 71.8% considered it acceptable.

This is also mirrored by answers given regarding general use of mobile devices in the context of health and illness. Again, data protection was the aspect about which the participants had the greatest reservations (113/213, 53.1%). They worried less about the technical reliability of the mobile devices (28/213, 13.1%) or susceptibility of the software (31/213, 14.6%). There were 11.7% (25/213) that were worried that the devices might be too complicated to use in a health context, most of these among those participants who did not yet own or have access to such devices (20/25).

## Discussion

### Principal Results

The physicians who participated in the survey mirror the widespread use of mobile devices; in this group, four out five (168/206, 81.6%) own a smartphone or tablet PC. In contrast, only every second patient (110/213, 51.6%) in our survey uses such a device, although the usage rate is equally high in the younger age groups, and only tapers off with increasing age of the participants, and especially for pensioners. Altogether, although addressing mobile smart devices in a broader sense, our findings for both groups mirror those of [21], where age and gender were strong predictors of advanced smartphone use in Germany, but also other European countries as well. And while one may argue that at least age wise, our sample of patients may not quite be representative of the overall population in Germany (with younger persons being somewhat underrepresented), due to our method of recruitment, we believe the participants to at least be representative for the typical patient population that doctors at a maximum care facility such as Hannover Medical School have to deal with in Germany.

Mobile devices are rarely used during direct contact between doctors and patients; rather, patients as well as physicians use them for looking up health related information or information about specific conditions. Although about half of the patients do not yet use a mobile device, the majority of patients would not object to doctors using mobile devices. Most of the patients have no problem with physicians using mobile devices to inform themselves about the patients’ condition, and about half of the physicians in our survey actually do so. Similarly, the vast majority of patients would have no objections to doctors using their device for patient education and providing or illustrating information, but in fact, less than 10% of physicians (20/206, 9.7%) use the devices for this purpose. Patients are more critical of doctors using their devices for storing and processing patient related data; every fifth patient has qualms about this, but approximately 5% of doctors (10/206, 4.8%) using a mobile device in the line of their work also use it for this purpose.

From our results, it is clear that the majority of patients do not have problems with doctors using mobile devices while caring for them. About one third of patients would like to be able to contact their doctors via electronic communication methods (email, chat, etc) using their smartphone or tablet PC. In view of the high rate of acceptance of mobile devices, the question arises whether the full potential of mobile technologies in physician-patient contacts, and the care process, is being realized. Problems with data protection and data security probably play an important role when it comes to explaining the slow progress in introducing mobile devices in this context. Both physicians as well as patients are worried about protection and security of sensitive patient data. Although one may suspect a seasonal effect on the answers, due to recent revelations about surveillance and cyber-espionage, data protection and privacy issues have always been on the political agenda.

**Figure 4:**
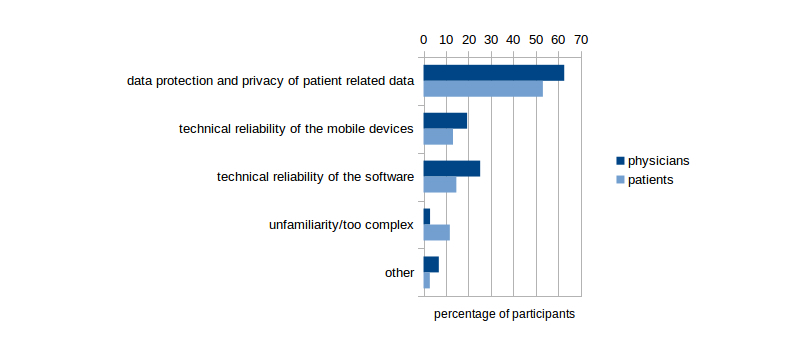
Concerns voiced by the participants about using mobile devices in a clinical setting.

Participants of both groups voiced only few concerns about the credibility of the content provided by the software or about the technical reliability of the devices (Figure 4 shows this information). There were only few doctors (17/206, 8.3%) who would refrain from using mobile devices during patient contact because they thought patients might be unfamiliar with or might not have access to such technology. All the same, 11.7% (25/213) of the surveyed patients expressed concerns that the use of smartphones and tablet PCs might be too complicated when it comes to health issues.

Our results support the claim that for patients, age and education play an important role in the use of mobile devices. They clearly show the digital divide that is so often mentioned in literature for older people, as well as educationally disadvantaged individuals [12,13]. Concerns about using mobile technologies in a medical context are often associated with whether patients are familiar with using mobile devices; those who own a mobile device clearly differ from those who do not have access to such a device. Still, another aspect also needs to be considered; access to and familiarity with computers with Internet access or mobile devices that also provide access to various (online) sources of information does not yet say anything about whether users can competently use this technology. Age, gender, and education are often mentioned as factors that contribute to either competent use or problems with such technologies, although this influence will probably lessen over time [12,13]. Considering the sociodemographic data available for the participating patients, it is clear that the majority of patients presenting at the central admissions point are of higher age, and there is also a considerable number of patients with lower and intermediate levels of education. Therefore, to fully realize the potential of mobile technologies in a health care context, one should include both the elderly as well as those who are educationally disadvantaged in all considerations, and implement measures to carefully introduce them to using these technologies in a safe manner.

For professional users, in spite of their prevalence, there has so far been little research supporting the use of mobile smart devices, a point also noted (albeit for emergency medicine) in [10]; available studies with similar aims regarding use of mobile devices by physicians are often either somewhat dated, at least considering the rapid developments in mobile technology [22-24], or they only consider a narrow angle [25,26].

Altogether, our findings, as well as the scarcity of literature on the matter, emphasize the need for further research into the use of mobile devices in medical settings, independent of which user group one considers, in order to fully realize the potentials mobile technologies can offer in medicine, while respecting users’ needs, hopes, and concerns.

### Conclusions

In conclusion, to ensure successful integration of mobile technologies in health care, in addition to expanding research, providers of medical content should be urged to closely check the requirements for their digital products, to ensure that they can be used in a safe manner, and to adapt their products to the needs of the specific users groups they target.

Our results suggest that the use of mobile devices during physician-patient contact will increase in the years ahead. Currently, only one out of five doctors consider it unlikely that he will use a mobile device for his work in the near future; for patients, usage rates will probably also increase as the younger generation, often called “digital natives”, moves up. Still, even though future patients and doctors will be increasingly familiar with mobile technologies, it is of utmost importance to educate them about how to safely use it.

## Multimedia Appendix 1

Relevant questions of the questionnaire used for the doctor's survey.

## Multimedia Appendix 2

Questionnaire used during the patient's survey.

## Abbreviations

apps: application software
PC: personal computer
