# Supplementary material for: Mobile Technologies: Expectancy, Usage, and Acceptance of Clinical Staff and Patients at a University Medical Center
Source: JMIR Mhealth Uhealth. 2014 Oct 21;2(4):e42. doi: 10.2196/mhealth.3799 (PMC4259908; doi:10.2196/mhealth.3799)
Supplement: Supplementary file 1 [file mhealth_v2i4e42_app1.pdf]

Table 3. Relevant questions of the questionnaire used for the doctors' survey.

#	Question	Possible answers
1	Are you currently using at least one mobile device that is able to run application software (apps)?	Yes / no
2	If yes, which device(s) are you using? (multiple answers allowed)	iPhone / iPad / iPod touch / Android Smartphone / Android Tablet / Blackberry / others
3	How did you obtain the device?	Private purchase or gift / provided by employer
4	How likely do you think it is that you will use a tablet (iPad, Android tablet or other tablet) in the line of your work in the coming years?	I already use one or more such devices at work / highly likely / somewhat likely / unlikely
5	If you already use a mobile device for your work, what are you using it for? (multiple answers allowed)	Electronic communication (email, chat, ...) / reference (medication, therapies) / researching literature / learning (eLearning, CME, ...) / diagnostic aid at a patient's bedside / aid for ordering procedures / for patient education or information / accessing patient records / decision support for ordering lab tests or medical imaging
6	What are other (additional) activities where you think mobile devices can be helpful during your work?	Free text answers
7	Which three apps (if any) are you using most in the line of your work?	Free text answers
8	What would keep you from using mobile apps during contact with your patients?	Concerns about the security of patient data / concerns about technical reliability of the devices / concerns about technical reliability of software / concerns about the hygiene of the devices /

		patients might be unacquainted with such devices / there is no possibility for reimbursement / no support by the employer / I am unfamiliar with such devices and would have to invest time to learn about them / I do not yet have a device and will probably not get one / other reasons
9	What would keep you from using mobile apps while collaborating with your colleagues?	Concerns about the security of patient data / concerns about technical reliability of the devices / concerns about technical reliability of software / concerns about the hygiene of the devices / patients might be unacquainted with such devices / no support by the employer / I am unfamiliar with such devices and would have to invest time to learn about them / I do not yet have a device and will probably not get one / other reasons
10	What is your professional function?	Chief physician / consultant / attending / junior doctor
11	What kind of department do you work in?	Patient wards / diagnostic or therapeutic (eg, radiology) / other medical department (eg, pathology) / other
12	How many years of professional experience do you have?	None / 1 up to 2 / 2 up to 4 / 4 up to 6 / 6 up to 10 / 10 up to 20 / 20 up to 30 / 30 and more
13	How old are you?	18-25 / 26-35 / 36-45 / 46-55 / 56 and older
14	Your gender?	Female / male
15	Do you have any additional remarks?	Free text answers
