# Supplementary material for: Mobile Technologies: Expectancy, Usage, and Acceptance of Clinical Staff and Patients at a University Medical Center
Source: JMIR Mhealth Uhealth. 2014 Oct 21;2(4):e42. doi: 10.2196/mhealth.3799 (PMC4259908; doi:10.2196/mhealth.3799)
Supplement: Supplementary file 2 [file mhealth_v2i4e42_app2.pdf]

**Table 4**. Questionnaire used during the patients' survey.

| # | Question | Possible answers |
|---|---|---|
| 1 | Smartphones and tablet PCs are handheld computers that can run application software, so-called "apps" that can be download from so-called "App Stores" available on the Internet. Are you currently using a smartphone and/or tablet PC? If no, continue with question 6. | Yes / no |
| 2 | What kind of smartphone or tablet PC are you using? (multiple answers allowed) | iPhone / iPad / iPod Touch / Android Smartphone / Android Tablet / Blackberry / Windows Tablet / Windows Smartphone / other |
| 3 | Are you currently using your smartphone or tablet PC to obtain or manage health related information or specific information for your condition? | Yes / no |
| 4 | For which health related activities do you use your device(s)? | Electronic communication (email, chat, ...) with my doctor / electronic communication with self-help groups / electronic communication with my insurer / electronic communication with other health care providers / finding doctors / finding drug stores / managing my health data (blood sugar, heart rate, blood pressure, lab values) / for obtaining information about symptoms and conditions / for obtaining information about diagnostic and therapeutic procedures / for finding my own diagnosis / for documenting and managing fitness related data (eg, using Runtastic) / other |

| | | |
|---|---|---|
| 5 | Health apps are software applications dealing with health related aspects. Some apps provide information about health issues, others aid in measuring or managing blood pressure; there are also some for reminding you about taking your medication or getting a diagnosis. How many health apps do you currently have on your smartphone / tablet PC? | None / 1-3 / 4-5 / 6-10 / more than 10 |
| 6 | Which health apps are you using regularly? | Free text answers |
| 7 | For which health related activities would you like to use your device(s)? | Electronic communication (email, chat, ...) with my doctor / electronic communication with self-help groups / electronic communication with my insurer / electronic communication with other health care providers / finding doctors / finding drug stores / managing my health data (blood sugar, heart rate, blood pressure, lab values) / for obtaining information about symptoms and conditions / for obtaining information about diagnostic and therapeutic procedures / for finding my own diagnosis / for documenting and managing fitness related data (eg, using Runtastic) / other |
| 8 | Would it be all right with you if your doctor were to use his smartphone or tablet PC for storing and processing your data? | Yes / no / don’t know |
| 9 | Would it be all right with you if your doctor were to use his smartphone or tablet PC to explain something to you? | Yes / no / don’t know |
| 10 | Would it be all right with you if your | Yes / no / don’t know |

| | | |
|---|---|---|
| | doctor were to use his smartphone or tablet PC for looking for information relevant to your condition? | |
| 11 | Which concerns do you have about using smartphones / tablet PCs in the context of your health / condition? (multiple answers allowed) | None / concerns about data security / concerns about the technical reliability of the devices / concerns about the reliability of the software / the devices are too complicated to use / other concerns |
| 12 | Which other concerns do you have? | Free text answers |
| 13 | Is your current stay at the hospital due to an illness lasting for more than 6 month? | Yes / no |
| 14 | How old are you (in years)? | 18-25 / 26-35 / 36-45 / 46-55 / 56-67 / 68 and older |
| 15 | What is your gender? | Female / male |
| 16 | What is your highest school leaving qualification? | Primary school / lower secondary school / intermediate secondary school / upper secondary school |
| 17 | Do you want to add anything, eg criticism, additional remarks, or do you have any suggestions for us? | Free text answers |
